# Ex Vivo Test of Complement Dysregulation in Atypical Hemolytic Uremic Syndrome Kidney Transplant patients: A Pilot Study

**DOI:** 10.1016/j.ekir.2023.10.003

**Published:** 2023-10-13

**Authors:** Caroline Duineveld, Romy N. Bouwmeester, Lambertus P.W.J. van den Heuvel, Nicole C.A.J. van de Kar, Jack F.M. Wetzels

**Affiliations:** 1 Department of Nephrology, Radboud University Medical Center, Radboud Research Institute, Nijmegen, The Netherlands; 2 Department of Pediatric Nephrology, Amalia Children’s Hospital, Radboud University Medical Center, Radboud Institute for Molecular Life Sciences, Nijmegen, The Netherlands

**Keywords:** atypical hemolytic uremic syndrome, biomarker, C5b-9 deposition, *ex vivo* complement assay, kidney transplantation, thrombotic microangiopathy

## Abstract

**Introduction:**

In 2014, a complement assay, which evaluates C5b-9 deposition on endothelial cells, was proposed as a biomarker for atypical hemolytic uremic syndrome (aHUS). Early diagnosis and/or prediction of aHUS (relapse) is pivotal in aHUS kidney transplant recipients who do not receive eculizumab prophylaxis.

**Methods:**

In this pilot study, serum samples of transplanted patients with aHUS in remission without eculizumab and patients with other primary kidney diseases (controls) were blinded and evaluated in the complement assay.

**Results:**

We included 13 patients with aHUS (4 males, 9 females) of median age of 54 years (range: 35–69) and median of 5.9 years (range: 0.25–14.1) after transplantation; and 13 controls (7 males, 6 females) of median age of 42 years (range: 27–60) and median of 5.8 years (range: 1.6–11.7) after transplantation. There were no significant differences in C5b-9 deposits between patients with aHUS and controls on resting cells (median of 136% [range: 93%–382%] and 121% [range: 75%–200%], respectively) and activated cells (median of 196% [range: 99%–388%] and 170% [range: 113%–260%], respectively). Three patients with aHUS and 4 controls showed elevated C5b-9 deposits on resting cells, which should correspond to active aHUS. None of these patients had laboratory signs of thrombotic microangiopathy (TMA). During follow-up (15.8 months, range: 6–21), estimated glomerular filtration rate remained stable in all. In 5 patients with aHUS with a genetic variant, no increase in C5b-9 deposits was found on activated endothelial cells, which contrasts with the literature suggesting that the test should identify carriers of a genetic variant.

**Conclusion:**

Our data question the routine use of the *ex vivo* complement assay in kidney transplant patients. Future studies should evaluate the test characteristics of assay in kidney transplant patients.

## Graphical abstract

**Figure:**
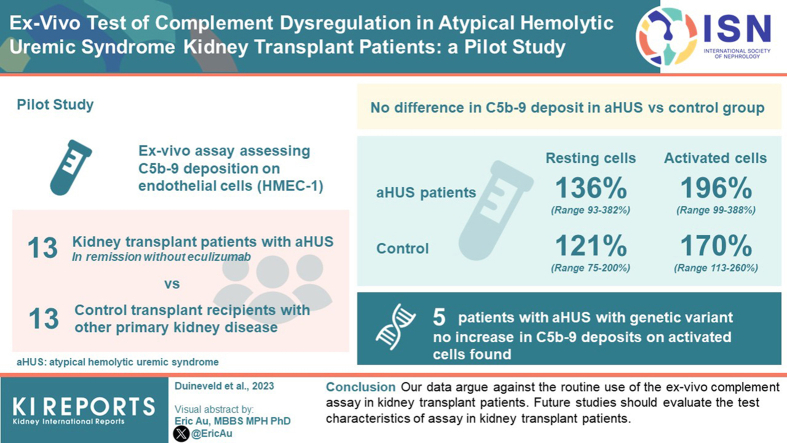

aHUS is a rare form of TMA caused by complement dysregulation. The diagnosis of aHUS is a diagnosis of exclusion. The combination of acute kidney injury, microangiopathic hemolytic anemia, and the presence of anti-CFH antibodies or a pathogenic mutation in one of the genes encoding for complement regulatory proteins strongly supports a diagnosis of aHUS. Although the introduction of the complement C5 inhibitor eculizumab improved outcome for patients with aHUS, some patients may still develop end-stage renal disease and are candidates for kidney transplantation.^1^ The risk of aHUS recurrence after kidney transplantation is considered high and therefore the use of prophylactic eculizumab therapy is recommended.^2^^,^^3^ Because of the high costs of eculizumab, in some countries eculizumab therapy is initiated reactively, at the time of suspected posttransplant aHUS recurrence.^4^ Using this strategy, early diagnosis of aHUS recurrence is important, as several studies showed that early start of eculizumab improved outcome.^5^^,^^6^ However, management of TMA after kidney transplantation is difficult. First, patients with aHUS may present with recurrence without laboratory signs of TMA and only slow loss of kidney function, which may result in a diagnostic delay.^4^^,^^7^, ^8^, ^9^, ^10^ Second, there are many other causes of TMA after kidney transplantation, which are difficult to discern from aHUS.^11^ There is currently no accurate diagnostic test for aHUS recurrence after kidney transplantation.

In 2014, Noris *et al.* developed an *ex vivo* complement assay to evaluate the presence of factors in serum that augmented complement activation on endothelial cells.^12^ In the initial study, the authors showed that serum of patients with active aHUS caused significantly higher C5b-9 deposition on both resting and activated endothelium. In patients with aHUS in remission, and in carriers of a genetic variant, significantly higher C5b-9 deposition was found on activated, but not on resting endothelium. C5b-9 deposition (both on resting and activated cells) normalized after treatment with eculizumab.^12^ In a subsequent study, it was suggested that the test would enable to identify patients who developed a relapse.^13^ To date, the studies evaluating the assay included mainly patients with native kidney disease (Supplementary Text S2). In this pilot study, we evaluated the C5b-9 deposition assay in kidney transplant patients with stable aHUS. We hypothesized to find elevated C5b-9 deposition on activated endothelial cells in all our patients with aHUS, because this assay was suggested to identify patients with an underlying complement dysregulation, with abnormal test results also documented in asymptomatic carriers.^14^ With respect to C5b-9 deposition on resting endothelial cells, we expected variable results: normal C5b-9 deposition should reflect absence of TMA activity whereas elevated C5b-9 deposition should herald TMA activity. We included a control group of kidney transplant patients, treated with similar immunosuppressive regimen including a calcineurin inhibitor, and a known native kidney disease diagnosis. We expected normal C5b-9 deposition on resting and activated endothelial cells in this control group.

## Methods

For this study, we collected serum samples of patients with aHUS who had received a kidney transplant and were not on eculizumab prophylaxis, thus at risk for relapse. All patients were diagnosed with aHUS before transplantation (based on laboratory and/or histological evidence of TMA, kidney injury and/or the presence of a genetic complement variant). After kidney transplantation, there had been no clinical evidence of aHUS recurrence. As controls, we used sera of kidney transplant patients with other primary kidney diseases. In addition, we collected sera of 2 kidney transplant patients with aHUS recurrence, in remission after treatment and withdrawal of eculizumab. We used serum of 1 patient with active aHUS relapse in the native kidneys as a positive control. The sera were stored at −80 °C and shipped (deeply frozen) to the Bergamo laboratory of Dr. M. Noris, Italy, for analysis. Serum-induced C5b-9 deposition was analyzed as previously described.^12^ In brief, immortalized human dermal microvascular endothelial cells (HMEC-1) are grown in confluence, and maintained in resting state or activated with adenosine 5’-diphosphate. Cells are incubated with patient or control serum, diluted (1:2) in test medium. Complement activation is quantitated after staining with antihuman C5b-9 antibodies and subsequent staining with a fluorescent secondary antibody. Fluorescent staining on endothelial cells surface was evaluated with the use of confocal microscopy. The area covered by fluorescent staining (thus C5b-9) was expressed as percentage compared to staining, using control serum pool. A positivity threshold of >150% (mean plus 2 SDs) was used, based on results of previous tests performed in healthy volunteers.^13^ The serum samples used in our study were analyzed in a blinded fashion (i.e., without knowledge of the clinical data).

Clinical data of the patients was collected from electronic patient records. Continuous variables were expressed as median and range. Patient characteristics and test results between the 2 groups were compared using independent sample *t*-test or Mann-Whitney U test in case of continuous data and chi-Square or Fischer’s Exact test in case of categorical variables. Correlation between 2 continuous variables was evaluated using Pearson correlation coefficient. *P* < 0.05 was considered to be statistically significant. Statistical analysis were performed using IBM SPSS Statistics (V.25.0:IBM; IBM, Armonk, NY) and figures were drawn using Graphpad Prism (V5.03; San Diego, CA).

The study was approved by the local Medical Ethical Committee. All patients gave written permission for participation in the study.

## Results

**Figure 1:**
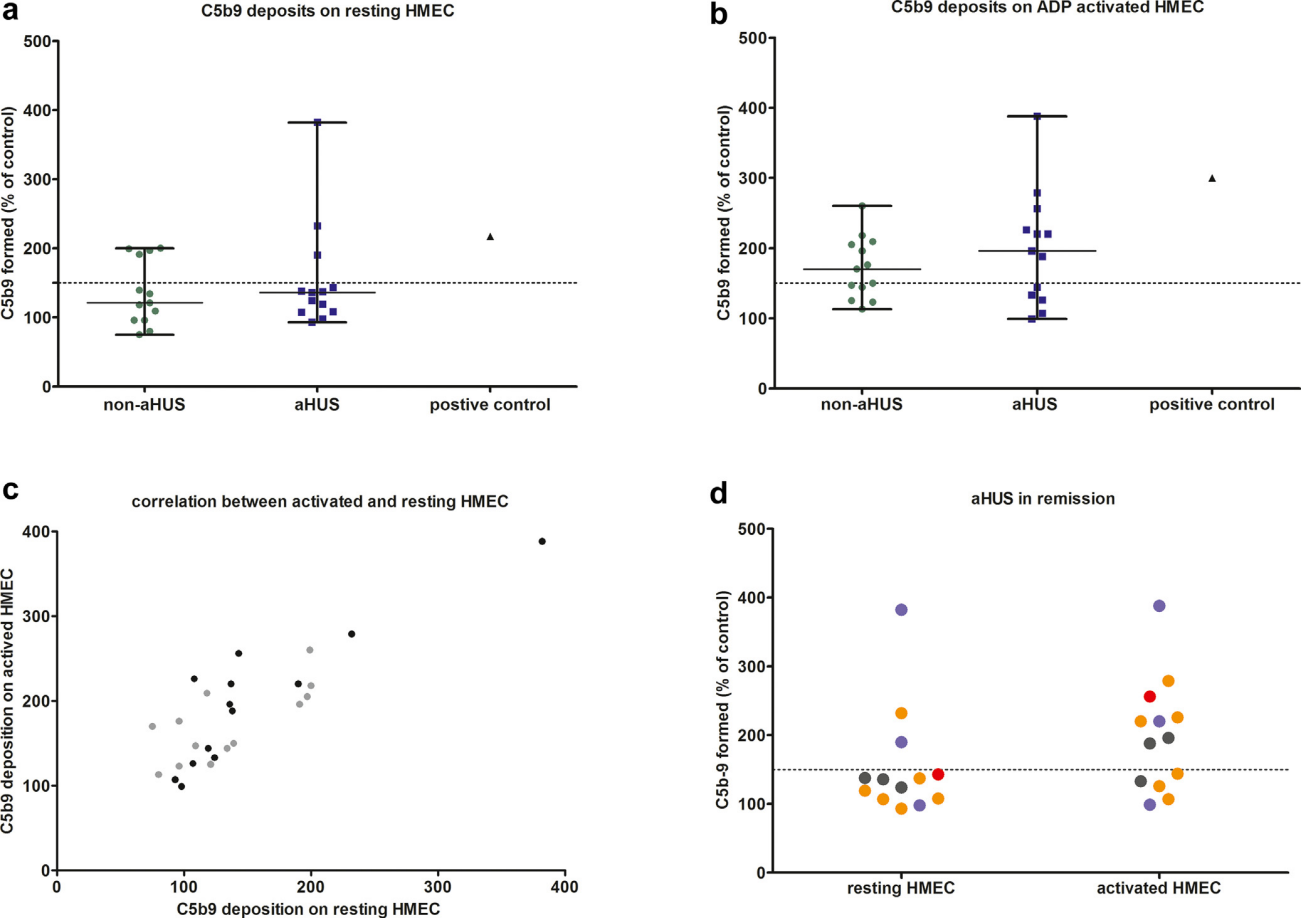
C5b-9 deposition in aHUS kidney transplant patients and non-aHUS kidney transplant patients. (a) C5b-9 deposition (individual values, median, range) on resting HMEC using sera of kidney transplant patients. The positive control is a patient with aHUS with active disease in native kidneys (patient A). (b) C5b-9 deposition (individual values, median, range) on activated HMEC using sera of kidney transplant patients. The positive control is a patient with aHUS with active disease in native kidneys (patient A). (c) correlation between C5b-9 deposition on resting and activated HMEC. Patients with aHUS are indicated in black and control patients in grey (*r* = 0.84, *N* = 26, *P* ≤ 0.0001). (d) C5b-9 deposition on resting and activated HMEC using serum of patients with aHUS, color-coded according to genotype. CFH variants are indicated in blue, C3 variants are indicated in orange, MCP variants are indicated in red. Patients with no variant found are indicated in black.

We included 13 patients with aHUS and 13 controls with other primary kidney disease. Characteristics of the controls (case 1–13) and the patients with aHUS (case 14–26) are given in Table 1. A description of the clinical history of the patients with aHUS can be found in the supplementary material (Supplementary Text S1). In the control group, the original kidney disease was verified by kidney biopsy, characteristic radiological findings, and/or genetic analysis. We found no significant differences in demography, transplant characteristics, and laboratory values between the 2 groups (Table 1). All patients had stable estimated glomerular filtration rate, and there were no laboratory signs of TMA (for individual values see Supplementary Tables S1–S3). Characteristics and test results of the positive control with active aHUS in native kidneys (patient A) and the 2 patients with known aHUS recurrence after kidney transplantation (patient B and C) are given in the supplementary material (Supplementary Figure S1). Results of the *ex vivo* assay are illustrated in Figure 1. There were no significant differences in C5b-9 deposition on resting or activated HMEC between the kidney transplant patients with stable aHUS and control patients (Figure 1a and b). Using resting endothelium, median values in transplant patients with aHUS were 136% (range: 93–382), and 121% (range: 75–200) in controls (*P* = 0.614). Using activated endothelium, median values in transplant patients with aHUS were 196% (range: 99–388), and 170% (range: 113–260) in controls (*P* = 0.705). Both in patients and controls, there were individuals with values that exceeded 150%. No significant differences were found in clinical or transplant characteristics, laboratory values or kidney function between patients with normal C5b-9 deposits and patients with elevated C5b-9 deposits on resting endothelium (Supplementary Table S4). Similarly, there were no significant differences in most characteristics between patients with normal or elevated C5b-9 deposits on activated endothelial cells, with the exception of a lower hemoglobin concentration in patients with elevated C5b-9 deposits (hemoglobin 7.7 mmol/l [6.6–8.7] vs. 8.4 mmol/l (7.3–10) (Supplementary Table S5). Of note, we observed a correlation between the degree of C5b-9 deposition on resting and activated endothelium, with no difference between the groups (Figure 1c).

**Table 1:** Patient characteristics

Variable	Non-aHUS patients, *N* = 13	aHUS patients, *N* = 13	*P*-value
Female gender, *n* (%)	6 (46.2%)	9 (69.2%)	0.428
Age (yrs)	42 (27–60)	54 (35–69)	0.120
Primary kidney disease (*n*)	Diabetic nephropathy, *n* = 2 IgA nephropathy, *n* = 4 ANCA vasculitis, *n* = 1 Cystinosis, *n* = 1 Fibrillary glomerulonephritis, *n* = 1 Membranous Nephropathy *n* = 1 Nephronophthisis, *n* = 1 Vesico-urethral reflux, *n* = 1 Autosomal dominant polycystic kidney disease, *n* = 1	Atypical HUS	n.a.
Number of previous kidney transplants	0 (0–2)	1 (0–2)	0.311
Genetic complement variant (n;%)	#	C3 variant 7 (53.8%) (R161W *n* = 6) CHF variant 3 (23.1%) MCP variant 1 (7.7%) No variant 2 (15.4%)	n.a.
Time since (last) kidney transplantation (yrs)	5.8 (1.6–11.7)	5.9 (0.25–14.1)	0.998
Type of donor, *n* (%)	Living donor, 11 (84.6%) Deceased donor, 2 (15.4%)	Living donor, 10 (76.9%) Deceased donor, 3 (23.1%)	1.000
HLA Mismatch	3 (2–6) (n=12)	4 (0–6) (n=13)	0.247
HLA antibodies positive, *n* (%)	5 (38.5%)	4 (30.8%)	0.500
DSA positive, *n* (%)	5 (38.5%)	3 (23.1%)	0.673
IS regimen at sample withdrawal (*n*)	Pred/TAC 7 Pred/TAC/MMF 4 Pred/TAC/AZA 1 TAC/MMF 1	Pred/TAC 3 Pred/TAC/MMF 6 Pred/TAC/AZA 1 TAC/AZA 1 TAC/MMF 1 Sirolimus/pred 1	0.590
Use of CNI, *n* (%)	13 (100%)	12 (92.3%)	1.000
TAC trough level at sample withdrawal (ug/l)	6.2 (3.1–10.3)	5.9 (4.5–7)	0.506
Patients with rejection, *n* (%)	3 (23.1%)	3 (23.1%)	1.000
Time since rejection (mos)	18.4 (18.0–106)	134.1 (45.1–168.5)	0.127
aHUS recurrence after last Ktx, *n* (%)	n.a.	0 (0%)	n.a.
Treatment with eculizumab, *n* (%)	n.a.	0 (0%)	n.a.
C5b-9 deposition on resting HMEC (%)	121.0 (75–200)	136.0 (93–382)	0.614
C5b-9 deposition on activated HMEC (%)	170.0 (113–260)	196.0 (99–388)	0.705
sCR (μmol/l)^a^	123.0 (77–169)	100.0 (76–176)	0.990
eGFR (ml/min per 1.73 m^2^)^a^	61.0 (37–92)	61.0 (28–86)	0.517
UPCR^a^ (g/10 mmol)	0.10 (0.08–0.46)	0.13 (0–0.86)	0.479
Hb^a^ (mmol/l)	8.2 (7–9)	7.4 (6.6–10)	0.057
Thrombocytes^a^ (x 10ꝰ/l)	205 (151–302)	206 (140–286)	0.975
LDH^a^ (u/l)	201 (165–256) (n=6)	206 (156–292) (n=11)	0.737
C3^a^ (mg/l)	983 (761–1359) (n=13)	972 (772–1220) (n=12)	0.851
C3d/C3 ratio^a^	5.1 (3–9.2) (n=13)	5.7 (3.1–10.1) (n=12)	0.230
Follow-up after sample collection (mos)	15.6 (6.2–18.6)	19.7 (10.8–21.1)	0.010
sCR at last follow-up (μmol/l)	127.0 (79–162)	107.0 (76–180)	0.792
eGFR (CKD-EPI) at last follow-up (ml/min per 1.73 m^2^)	59.0 (39–96)	57.0 (26–84)	0.594
UPCR at last follow-up (g/10 mmol)	0.10 (0.05–0.86)	0.14 (0.08–0.69)	0.186

aHUS, atypical hemolytic uremic syndrome; Hb, hemoglobin; CNI, calcineurin inhibitors; eGFR, estimated glomerular filtration rate (CKD-EPI formula); Ktx, kidney transplantation; LDH, lactate dehydrogenase (normal <250 u/l); n.a., not applicable; sCr, serum creatinine; TAC, tacrolimus trough level; UPCR, urine protein creatinine ratio; DSA, donor-specific antibody

Results are given as median (range).

a Data obtained at the time of sample collection.

# in 4 control patients with elevated C5b-9 deposition on resting HMEC genetic analysis was performed: in one patient a benign CFB variant was found.

There were 3 patients with aHUS and 4 control patients with elevated C5b-9 deposits on resting endothelium, which according to the literature could herald aHUS activity. All 7 patients were followed-up with for median of 15.8 months (range: 6.2–21.1). During follow-up, no patient developed laboratory features of TMA. Graft function in these 7 patients has remained stable with an estimated glomerular filtration rate of 60 ml/min per 1.73 m^2^ (range: 33–86) at last follow-up versus 61 ml/min per 1.73 m^2^ (range: 34–88) at sample collection. In addition, no patient developed increasing proteinuria. Of note, all 3 patients with aHUS were vaccinated against COVID during follow-up, which did not result in aHUS recurrence. Because it was suggested that elevated C5b-9 deposition on activated (but not on resting) endothelium was associated with aHUS carrier phenotype, we further evaluated the test results. In the control group, there were 7 patients with elevated C5b-9 deposits on activated HMEC. These patients were diagnosed with the following primary kidney diseases: vesico-urethral reflux (*n* = 1), IgA nephropathy (*n* = 3), membranous nephropathy (*n* = 1), autosomal dominant polycystic kidney disease (*n* = 1), and fibrillary glomerulonephritis (*n* = 1). Four of them (case 4, 5, 6, and 8) also showed elevated C5b-9 deposits on resting HMEC. In these 4r patients, a screening for complement gene variants was performed. No pathogenic variants were found: in 1 patient, a heterozygous complement factor B variant (c.1697A>C, p.Glu566Ala) was detected, which was classified as benign.^15^ Notably, in 5 patients with aHUS with a genetic variant the test on activated HMEC was negative.

## Discussion

The introduction of eculizumab as effective therapy in patients with aHUS has stimulated the search for biomarkers that would allow early and accurate diagnosis. The biomarker should allow to establish a diagnosis of aHUS at presentation; however, preferably it should also predict aHUS recurrence after eculizumab withdrawal. Clinically, such a test would be most valuable in kidney transplant recipients, who are not treated with eculizumab prophylaxis. In 2014, the *ex vivo* complement deposition assay was proposed as biomarker for patients with aHUS. To date, the majority of the patients evaluated with this assay were patients with native kidney disease. We hypothesized that the endothelial cell assay could also be of value in the kidney transplant population. Although our study is small, our data clearly show that test results in kidney transplant recipients are heterogeneous, and not according to our initial hypothesis. In some patients with aHUS, we observed elevated C5b-9 deposition on resting cells, and although we cannot formally exclude the presence of subtle TMA in kidney biopsy, kidney graft function has been stable over a follow-up period of more than 18 months, thereby arguing against clinically important disease activity. In our view, these data do not support the use of eculizumab based on test results only. Most importantly, abnormal test results were also found in control patients. Follow-up indicated that these patients also had stable graft function. Although theoretically, the control patients might carry genetic variants in complement genes, this seemed unlikely in view of the rarity of such variants.^16^ Moreover, genetic analysis was performed in 4 control patients with elevated C5b-9 deposition on resting and activated endothelium, and no pathogenic genetic variants were detected.

The C5b-9 assay is currently being used in several centers and patient samples can be analyzed upon request. Our results caution against the routine use of the endothelial assay in the management of kidney transplant patients with aHUS. In the clinic, we would need a robust and specific test. Our data suggest low specificity (i.e., high number of false positive test results). Admittedly, these results were obtained using reference values obtained in a cohort of healthy controls.^13^ Different reference values might be needed in a kidney transplant population and may be dependent on transplant related factors such as immunosuppressive regimen, presence of donor-specific antibodies, humoral or cellular rejection, BK viremia, and others. Further studies are necessary. Suitable reference values should be established from stable kidney transplant patients without a complement-related disease in the native kidneys. In addition, *in vitro* studies should be performed to assess if immunosuppressants alter test results. The test should be evaluated in larger prospective studies, which should include aHUS transplant recipients with recurrence (clinically or biopsy-proven) and patients who are in remission (biopsy-proven, to exclude low grade renal-limited TMA). Ideally, serial samples should be taken from patients with aHUS before transplantation and afterward to allow correlation with clinical status.

Although our findings may seem contradictory to the reported literature data, a critical evaluation of the studies supports the view that a positive endothelial cell assay on resting endothelium is also not highly specific for a diagnosis of aHUS in patients with TMA in native kidneys. A positive test is often observed in clinical conditions that are characterized by complement activation (secondary TMA).^13^^,^^17^ Although many authors emphasize the possible benefits of eculizumab in secondary TMA, we feel that more evidence is needed. In this respect, a recent randomized controlled trial in patients with STEC-HUS showed that eculizumab therapy did not improve primary outcome (renal replacement therapy duration) and did not result in faster resolution of hematological signs of TMA.^18^ A detailed discussion on native kidney TMA is beyond the scope of this manuscript; however, it can be found in the supplementary appendix (Supplementary Text S2, Supplementary Tables S6–S9).

Of note, we observed normal C5b-9 deposition on activated endothelial cells in 5 patients with aHUS and a genetic variant. We have no easy explanation of these findings, which are in contrast to the literature data, suggesting that a positive test on activated endothelium allows to detect patients with a genetic predisposition to aHUS.^14^ All but 1 patient had a C3 mutation. Interestingly, the association between a positive test on activated endothelium and carriership of an aHUS genetic mutation might be dependent on polymorphisms. Indeed, it was suggested that a positive assay was found in carriers of an MCP mutation, but only in individuals who concomitantly were carriers of at least 1 CFH risk haplotype.^13^^,^^14^ Further studies are needed to assess which factors influence the *ex vivo* endothelial test results: genetic variants, risk haplotypes, and/or other (yet unknown) factors.

In conclusion, development of a diagnostic and prognostic biomarker for kidney transplant patients with aHUS is of utmost importance. Unfortunately, our data show heterogenous results of the C5b-9 assay in kidney transplant patients. At this moment, we caution against the routine use of the C5b-9 assay in guiding management of kidney transplant patients with aHUS. Well-designed prospective studies are needed to evaluate the role of the assay as biomarker.

## Disclosure

NvdK and JW received grant support from the Dutch Board of Health Insurance Companies. NvdK received consultancy fees from Alexion, Roche Pharmaceuticals, and Novartis; and is subinvestigator in APL2-C3G trial, Apellis. JW received consultancy fees from Alexion and Novartis. CD is sub-investigator in the APL2-G3G trial, Apellis. JW and NvdK are members of the European Reference Network for Rare Kidney Diseases (ERKNet)-Project No 739532. All remaining authors declare no conflicting interest.

## Supplementary Material

Supplementary File (PDF)

**Text S1.** Clinical history of the patients with aHUS.

**Text S2.** Review of the literature on the C5b-9 endothelial cell assay.

**Figure S1.** C5b-9 deposition in patients with active Ahus.

**Table S1.** Clinical characteristics of the controls (1–13) and patients with aHUS (14–26).

**Table S2.** Laboratory characteristics at time of sample withdrawal and last follow-up of the controls (1–13) and patients with aHUS (14–26).

**Table S3.** Biopsy results of all biopsies taken during study period.

**Table S4.** Characteristics at sample withdrawal of patients with and without elevated C5b-9 deposition on resting HMEC.

**Table S5.** Characteristics at sample withdrawal of patients with and without elevated C5b-9 deposition on activated HMEC.

**Table S6.** Clinical characteristics of patients with active aHUS at disease onset and results of the C5b-9 assay.

**Table S7.** Results of C5b-9 assay on resting endothelium in patients with secondary TMA.

**Table S8.** Comparison of clinical diagnosis and diagnosis based on C5b-9 assay as reported by Timmermans *et al.*

**Table S9.** Clinical parameters of the 5 patients with aHUS reported by Galbusera *et al.* at the time of elevated C5b-9 deposition on resting HMECs.
