# Supplementary material for: Ex Vivo Test of Complement Dysregulation in Atypical Hemolytic Uremic Syndrome Kidney Transplant patients: A Pilot Study
Source: Kidney Int Rep. 2023 Oct 13;9(1):145–51. doi: 10.1016/j.ekir.2023.10.003 (PMC10831345; doi:10.1016/j.ekir.2023.10.003)
Supplement: Supplementary File (PDF) [file mmc1.pdf]

Supplementary material

Supplementary Figure S1: C5b-9 deposition in patients with active aHUS

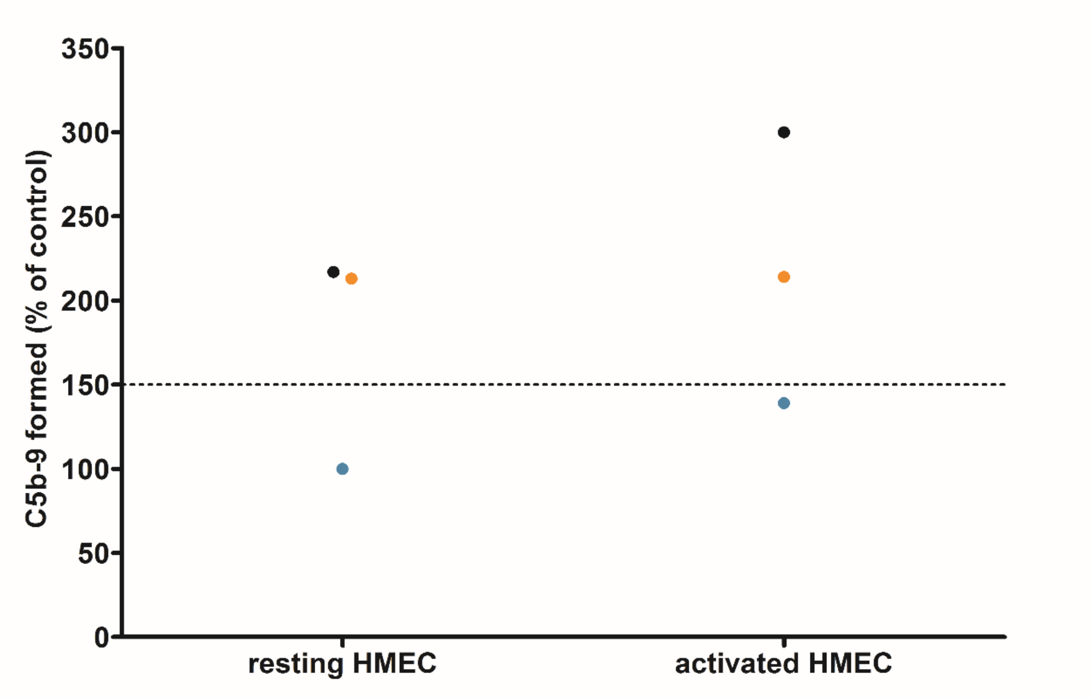

Legend:

Patient A (used as positive control, and indicated in black) presented with a relapse of aHUS in the **native kidneys**. This male patient carried a pathogenic C3 variant (p.Arg161Trp) and was treated with eculizumab for a first aHUS episode at age of 32 years. One year after eculizumab withdrawal he presented with AKI (increase in serum creatinine from 92 to 166 µmol/L) and laboratory evidence of TMA (thrombocytes 73x10^9/L, LDH 788 IU/L, haptoglobin not measured). Eculizumab therapy resulted in TMA remission and improvement of kidney function to pre-treatment values. The sample used for the ex-vivo

complement assay was taken at the time of presentation with relapse, before re-start of eculizumab, and was used as a positive control. C5b-9 deposition on resting cells was 217%, on activated cells 300%.

Patient B (indicated in orange) was known with ESRD due to aHUS. This male patient carried a pathogenic C3 variant (p.Arg161Trp). He received a kidney transplant and developed aHUS recurrence 4.5 months after transplantation. He was treated with eculizumab resulting in recovery of TMA parameters and improvement of kidney function. The sample used for the ex-vivo complement assay was taken 23.0 months after discontinuation of eculizumab during a period of clinical remission, with stable eGFR and no laboratory signs of TMA. The sample showed elevated C5b-9 deposition on both resting and activated endothelium (respectively 213%, and 214%). Seven months after sample withdrawal, kidney function deteriorated after an pneumocystis pneumonia, without laboratory sign of TMA. A kidney biopsy showed signs of rejection (mild glomerulitis, tubulitis and peritubular capillaritis, with a negative C4d staining, and glomerular thrombosis. The patient was treated with methylprednisolone and eculizumab was re-started resulting in improvement of kidney function.

Patient C (indicated in blue) was known with ESRD due to aHUS. This female patients carried a CFH variant (p.Tyr475His), which was classified as variant of unknown significance. She received a kidney transplantation and developed a aHUS recurrence 43.5 months after kidney transplantation during pregnancy. She was treated with eculizumab resulting in recovery of TMA parameters and improvement of kidney function. The sample used for the ex-vivo complement assay was taken 25.4 months after discontinuation of eculizumab during a period of clinical remission, with stable eGFR and no laboratory signs of TMA. The sample showed normal levels of C5b-9 deposition on both resting and activated endothelium (respectively 100% and 139%). During follow-up (20.7 months) after sample withdrawal no aHUS relapse was observed.

Supplementary Table S1: Clinical characteristics of the controls (1-13) and aHUS patients (14-26)

Case	Age (years)	Gender	Primary kidney disease	Genetic variant	CFH haplotype *	Number of previous Ktx	Interval Ktx to sample withdrawal (years)	Type of donor	MM	HLA Ab	DSA's	IS	TAC trough level (ug/L)
**1**	60	M	Diabetic nephropathy (type 1)	ND	ND	0	10.5	LURD	3	Negative	Negative	pred, TAC	7.4
**2**	27	M	Cystinosis	ND	ND	1	7.9	LRD	2	Class I and II	Positive	pred, TAC	5.7
**3**	35	M	Diabetic nephropathy (type 1)	ND	ND	0	2.8	LRD	2	Negative	Negative	TAC, MMF	10.3
**4**	41	M	IgAN	Negative	H3/-	0	6,0	LRD	3	Negative	Negative	pred, TAC	5.4
**5**	55	M	IgAN	CFB (p.Glu566Ala) Benign	H3/-	0	11.7	LURD	6	Negative	Negative	pred, TAC	7.4
**6**	56	M	MN	Negative	-/-	1	5.8	LRD	3	Negative	Negative	pred, TAC	6.2
**7**	40	M	VUR	ND	ND	0	11.2	LRD	2	Negative	Negative	pred, TAC	6.4
**8^**	60	F	Fibrillary glomerulonephritis	Negative	-/-	0	1.6	LRD	3	Class II	Positive	pred, TAC, MMF	6.1
**9**	53	F	IgAN	ND	ND	2	2.2	HBD	3	Class I and II	Positive	pred, TAC, MMF	6.4
**10**	57	F	ADPKD	ND	ND	0	5.8	LURD	4	Class I and II	Positive	pred, TAC, MMF	7.2

**11**	33	F	Nephronophthisis	ND	ND	1	4.8	DCD	2	Negative	Negative	pred, TAC	4.4
**12^**	27	F	ANCA vasculitis	ND	ND	0	3.5	LRD	3	Class I and II	Postitive	pred, TAC, MMF	3.1
**13^**	42	F	IgAN	ND	ND	0	8.9	LRD	2	Negative	Negative	pred, TAC, AZA	4.0
**14**	43	M	aHUS	CFH (p.Arg53His) Likely pathogenic	-/-	1	0.25	DBD	3	Negative	Negative	pred, TAC, MMF	6.7
**15**	53	F	aHUS	Negative	-/-	0	1.41	LURD	4	Negative	Negative	pred, TAC	6.3
**16^**	69	F	aHUS	C3 (p.Arg161Trp) Pathogenic	H3/-	0	3.84	LURD	5	Class II	Positive	pred, TAC	4.5
**17**	39	F	aHUS	CFH (p.Gln950His) Likely benign	-/-	0	3.93	LURD	4	Negative	Negative	pred, TAC, AZA	5.5
**18**	54	F	aHUS	C3 (p.Arg161Trp) Pathogenic	-/-	2	5.14	DBD	0	Class I and II	Negative	pred, TAC, MMF	6.5
**19**	42	F	aHUS	Negative	H3/-	1	5.33	DBD	3	Class I and II	Positive	pred, TAC, MMF	5.2
**20**	69	F	aHUS	C3 (p.Arg161Trp) Pathogenic	H3/-	2	5.89	LURD	5	Negative	Negative	pred, TAC, MMF	5.3
**21**	65	M	aHUS	C3 (p.Arg161Trp) Pathogenic	ND	1	6.92	LURD	3	Negative	Negative	pred, TAC	4.9
**22**	55	M	aHUS	C3 (p.Lys65Gln) Pathogenic	ND	1	8.51	LURD	6	Negative	Negative	pred, TAC, MMF	4.7
**23**	38	F	aHUS	CFH (p.Arg1210Cys) Pathogenic	H3/-	1	8.63	LRD	2	Class II	Positive	pred, TAC, MMF	6.2
**24**	64	F	aHUS	C3 (p.Arg161Trp)	H3/-	0	9.43	LURD	4	Negative	Negative	TAC, AZA	7.0

				Pathogenic									
**25^**	61	M	aHUS	MCP (p.Asp271_Ser272del) Pathogenic	H3/H3	0	11.24	LURD	6	Negative	Negative	TAC, MMF	6.4
**26^**	35	F	aHUS	C3 (p.Arg161Trp) Pathogenic	ND	0	14.14	LRD	1	Negative	Negative	sirolimus, pred	6.5#

#Sirolimus level

^Patients with a biopsy proven rejection

*The risk haplotype CFH-H3 consist of homozygosity for c.-331C>T (rs3753394), c.2016A>G (rs3753396), and c.2808G>T (rs1065489).

Abbreviations: ADPKD: autosomal dominant kidney disease, aHUS: atypical hemolytic uremic syndrome, AZA: azathioprine, DBD: donation after brain death, DCD: donation after cardiac death, DSA: donor specific antibodies, F: female, HLA ab: anti-human leukocyte antigen antibodies, IgAN: IgA nephropathy, IS: immunosuppression, Ktx: kidney transplantation, LRD: living related donor, LURD: living unrelated donor, M: male, MMF: mycophenolate mofetil, MN: membranous nephropathy, n.a.: not applicable, ND: not done, pred: prednisolone, TAC: tacrolimus, VUR: vesicoureteral reflux.

Supplementary Table S2: Laboratory characteristics at time of sample withdrawal and last follow-up of the controls (1-13) and aHUS patients (14-26)

Case	Hb (mmol/L)	Platelets ($x10^9$/L)	LDH (U/L)	sCr (µmol/L)	UPCR (g/10mmol)	C3 (mg/L)	C3d/C3 ratio	C5b-9 on resting HMEC (%)*	C5b-9 on activated HMEC (%)*	FU duration after sample withdrawal (months)	sCr at last FU (µmol/L)	UPCR at last FU (g/10mmol)
**1**	9	151	Not done	169	0.19	761	4.6	96	123	13.8	160	0.09
**2**	9	181	Not done	97	0.11	839	3.0	139	150	14.7	93	0.11
**3**	8.1	209	217	123	0.10	1077	6.7	109	147	14.7	127	0.09
**4**	8	247	256	125	0.10	1359	5.6	**200**	**218**	15.3	128	0.07
**5**	8.7	176	185	85	0.10	1027	4.2	**191**	**196**	15.8	86	0.05
**6**	8.2	226	222	148	0.10	965	4.2	**197**	**205**	15.8	162	0.16
**7**	8.2	159	171	126	0.13	1104	5.1	75	**170**	18.1	133	0.17
**8^**	7	280	Not done	93	0.15	1205	5.5	**199**	**260**	6.2	90	0.6
**9**	8.2	205	Not done	77	0.08	938	5.4	96	**176**	15.4	79	0.07
**10**	8.2	239	Not done	101	0.08	1014	4.0	118	**209**	15.6	100	0.09
**11**	8	302	Not done	87	0.10	881	9.2	134	144	16.5	80	0.10
**12^**	8.2	189	165	127	0.12	983	4.1	121	125	16.6	128	0.86
**13^**	8.7	167	Not done	135	0.46	897	5.4	80	113	18.6	135	0.2
**14**	6.6	211	292	100	0.4	965	4.6	**190**	**220**	10.8	107	0.33
**15**	8.3	184	177	92	0.06	1039	3.1	136	**196**	20.4	101	0.08
**16^**	8.7	181	Not done	161	0.43	979	7.6	119	144	8.8	169	0.2
**17**	7.3	216	156	78	0.07	1211	3.1	98	99	17.2	76	0.09
**18**	6.9	140	161	99	0.86	834	10.1	108	**226**	16.3	84	0.56
**19**	7.7	234	200	93	0.18	1220	3.7	138	**188**	18.4	84	0.14
**20**	8.4	197	255	83	0.05	1086	5.2	93	107	20.0	94	0.11
**21**	7.2	202	206	176	0.13	840	9.2	**232**	**279**	16.3	180	0.55

**22**	10	174	221	115	0.1	860	6.2	124	133	20.0	108	0.12
**23**	7.4	253	194	76	0.13	925	5.2	**382**	**388**	21.1	81	0.10
**24**	7.8	254	208	154	0.14	Not done	Not done	107	126	11.4	126	0.28
**25^**	7.2	286	Not done	129	0.10	772	9.2	143	**256**	21.1	117	0.10
**26^**	7	206	233	135	0.55	1172	6.6	137	**220**	21.1	132	0.69

*The bold printed values are above upper limit of normal

^Patients with a biopsy proven rejection

Abbreviations: FU: follow-up, Hb: hemoglobin, LDH: lactate dehydrogenase (normal <250 u/L), sCr: serum creatinine, UPCR: urine protein-creatinine ratio

Supplementary Table S3: Biopsy results of all biopsies taken during study period

Case	Number of biopsy's taken	Biopsy results	Rejection	Anti-rejection treatment	Timing of Bx	Thrombosis in Bx
**3**	1	Bx1: no rejection, C4d negative, tubulopathy, mild arteriolar hyalinosis	No	No	Bx1 was taken 32 months before sample withdrawal	No
**8**	2	Bx1: TCMR 2a and ABMR, C4d positive, tubulopathy, mild arteriolar hyalinosis, no chronic damage<br>Bx2: TCMR 1, possible ABMR, C4d negative, tubulopathy, mild arteriolar hyalinosis, 10% IFTA	TCMR and ABMR	Methylprednisolone after Bx1 and after Bx2	Bx2 was taken 18 months before sample withdrawal	No
**12**	3	Bx1: TCMR type 1, C4d negative, severe arteriolar hyalinosis with remnants of thrombosis, 30% IFTA<br>Bx2: TCMR and ABMR, C4d positive, tubulopathy, severe arteriolar hyalinosis, 30-40% IFTA<br>Bx3: chronic TCMR and AMBR, C4d positive, tubulopathy, severe arteriolar hyalinosis, 30% IFTA	TCMR and AMBR	Methylprednisolone after Bx1 and after Bx2	Sample was withdrawn 18 months after Bx1 and 7 months before Bx2	Yes, in Bx1
**13**	4	Bx1: no rejection, C4d negative, severe arteriolar hyalinosis<br>Bx2: TCMR type 2a, C4d negative, tubulopathy, mild arteriolar hyalinosis, mild chronic damage<br>Bx3: TCMR type 2a, C4d negative, tubulopathy, moderate arteriolar hyalinosis with remnants of thrombosis, mild chronic damage<br>Bx4: no rejection, C4d negative, tubulopathy, moderate arteriolar hyalinosis, mild chronic damage	TCMR	Methylprednisolone before Bx1 and after Bx2	Bx4 was taken 101 months before sample withdrawal	Yes, in Bx3
**16**	3	Bx1: ABMR, C4d positive, tubulopathy, endothelial swelling and arteriolar hyalinosis without thrombosis, 10-20% IFTA<br>Bx2: no rejection, C4d negative, no hyalinosis or tubulopathy, 5% IFTA<br>Bx3: BK nephropathy, severe tubulopathy, no rejection, C4d negative, no TMA	ABMR	Methylprednisolone, alemtuzumab after Bx1	Bx3 was taken 40 months before sample withdrawal	No
**21**	1	Bx1: BK nephropathy, no rejection, C4d negative, no TMA, no chronic damage	No	No	Bx1 was taken 79 months before sample withdrawal	No

**25**	2	Bx1: tubulopathy, no rejection, C4d negative, no TMA, no chronic damage<br>Bx2: TCMR type 2a, C4d negative, severe tubulopathy, mild arteriolar hyalinosis, no chronic damage	TCMR	Methylprednisolone, ATG after Bx2	Bx2 was taken 134 months before sample withdrawal	No
**26**	1	Bx1: TMCR type 2a, C4d negative, no chronic damage	TCMR	Methylprednisolone, ATG after Bx1	Bx1 was taken 168 months before sample withdrawal	No

Abbreviations: ABMR: antibody-mediated rejection, ATG: anti-thymocyte globulin, Bx: biopsy, IFTA: interstitial fibrosis and tubular atrophy, TCMR: T-cell mediated rejection.

Supplementary Table S4: Characteristics at sample withdrawal of patients with and without elevated C5b-9 deposition on resting HMEC

Variable	Elevated C5b-9 on resting HMEC (n=7)	Normal C5b-9 on resting HMEC (n=19)	P value
**Female gender (%)**	2/7 (28.6%)	13/19 (68.4%)	0.095
**Age (years)**	55 (38-65)	53 (27-69)	0.605
**Primary kidney disease aHUS**	3/7 (42.9%)	10/19 (52.6%)	1.000
**Previous transplantation**	4/7 (57.1%)	7/19 (36.8%)	0.407
**Deceased donor transplantation**	1/7 (14.3%)	4/19 (21.1%)	1.000
**HLA antibodies positive**	2/7 (28.6%)	7/12 (58.3%)	1.000
**DSA positive**	2/7 (28.6%)	6/19 (31.6%)	1.000
**Rejection episode**	1/7 (14.3%)	5/19 (26.3%)	1.000
**Time interval since transplantation (years)**	6.0 (0.25-11.7)	5.8 (1.4-14.1)	0.579
**Elevated C5b-9 on activated HMEC**	7/7	8/19	0.010
**sCr (µmol/L)**	100 (76-176)	115 (77-169)	0.996
**eGFR (ml/min/1.73m²)**	61 (34-88)	61 (28-92)	0.401
**UPCR (g/10 mmol)**	0.13 (0.1-0.4)	0.11 (0.05-0.86)	0.778
**Hb (mmol/L)**	7.4 (6.6-8.7)	8.2 (6.9-10)	0.103
**Thrombocytes (x10⁹/L)**	226 (176-280)	189 (140-302)	0.204
**C3 (mg/L)**	965 (840-1359)	979 (761-1220)	0.397
**TAC trough levels (ug/L)**	6.2 (4.9-7.4)	5.7 (3.1-10.3)	0.732
**sCR at last FU (µmol/L)**	107 (81-180)	108 (76-169)	0.599

Continuous variables are indicated in median (range)

Abbreviations: aHUS: atypical hemolytic uremic syndrome, DSA: donor specific antibodies, eGFR: estimated glomerular filtration rate (CKD-EPI formula), FU: follow-up, Hb: hemoglobin, HLA: human leukocyte antigen, HMEC: human microvascular endothelial cells, sCr: serum creatinine, TAC: tacrolimus, UPCR: urine protein-creatinine ratio.

Supplementary Table S5: Characteristics at sample withdrawal of patients with and without elevated C5b-9 deposition on activated HMEC

Variable	Elevated C5b-9 on activated HMEC (n=15)	Normal C5b-9 on activated HMEC (n=11)	P value
**Female gender (%)**	8/15 (53.3%)	7/11 (63.6%)	0.701
**Age (years)**	53 (35-65)	42 (27-69)	0.572
**Primary kidney disease aHUS**	8/15 (53.3%)	5/11 (45.5%)	1.000
**Previous transplantation**	7/15 (46.7%)	4/11 (36.4%)	0.701
**Deceased donor transplantation**	4/15 (26.7%)	1/11 (9.1%)	0.614
**HLA antibodies positive**	6/15 (40%)	3/11 (27.3%)	0.683
**DSA positive**	5/15 (33.3%)	3/11 (27.3%)	1.000
**Rejection episode**	3/15 (20%)	3/11 (27.3%)	1.000
**Time interval since transplantation (years)**	5.8 (0.25-14.1)	5.9 (2.8-10.5)	0.971
**Elevated C5b-9 on resting HMEC**	7/15	0/11	0.010
**sCr (µmol/L)**	100 (76-176)	123 (78-169)	0.388
**eGFR (ml/min/1.73m²)**	61 (34-88)	62 (28-92)	0.310
**UPCR (g/10 mmol)**	0.13 (0.06-0.86)	0.11 (0.05-0.46)	0.838
**Hb (mmol/L)**	7.7 (6.6-8.7)	8.4 (7.3-10.0)	0.006
**Thrombocytes (x10^9/L)**	211 (140-286)	189 (151-302)	0.393
**C3 (mg/L)**	1014 (772-1359)	938 (761-1211)	0.288
**TAC trough levels (ug/L)**	6.4 (4.9-7.4)	5.3 (3.1-10.3)	0.296
**sCR at last FU (µmol/L)**	101 (79-180)	126 (76-169)	0.578

Continuous variables are indicated in median (range)

Abbreviations: aHUS: atypical hemolytic uremic syndrome, DSA: donor specific antibodies, eGFR: estimated glomerular filtration rate (CKD-EPI formula), FU: follow-up, Hb: hemoglobin, HLA: human leukocyte antigen, HMEC: human microvascular endothelial cells, sCr: serum creatinine, TAC: tacrolimus, UPCR: urine protein-creatinine ratio.

Supplemental Text S1: Clinical history of the aHUS patients

Patient 14 presented at the age of 35 years old with severe hypertension, hypertensive retinopathy, left ventricular hypertrophy (on cardiac ultrasound) and ESRD. The patient had no previous medical history. Relevant laboratory values at presentation were: serum creatinine 1465 µmol/L, Hb 3.6 mmol/L, thrombocytes 219 $x10^9$/L, LDH 956 u/L, haptoglobin 0.34 g/L, schistocytes positive, proteinuria 1.24 gram/24 hours. Auto-immune serology was negative. An ultrasound showed a right kidney of 12-13 cm and a left kidney of 10 cm. A renal artery stenosis was excluded with angiography. A kidney biopsy showed chronic and active TMA, with 30% glomerulosclerosis and moderate IFTA. The patient started on hemodialysis and intensive blood pressure treatment. Genetic diagnostics revealed a CFH variant. Five years after presentation the patient received a first (LUR donor) kidney transplant which was lost due TCMR, which was followed by BK nephropathy. A second (deceased donor) kidney transplantation was performed two years later. The blood sample for evaluation of C5b-9 deposition was taken 3 months after this second kidney transplantation.

Patient 15, with a positive family history for aHUS, presented at the age of 43 years with acute kidney injury and TMA. Relevant laboratory values at presentation were: serum creatinine 384 µmol/L, thrombocytes 120 $x10^9$/L, LDH 1979 u/L. She was treated with plasmapheresis with limited effect. Extensive genetic analysis was performed but no variant was found. One year after presentation hemodialysis was started. After 8 years of hemodialysis a kidney transplantation (LUR donor) was performed. The blood sample for evaluation of C5b-9 deposition was taken 1.4 years after the transplantation.

Patient 16, with a positive family history for aHUS, presented at the age of 62 years with progressive CKD and TMA. Relevant laboratory values at presentation were: serum creatinine 268 µmol/L, thrombocytes 81 $x10^9$/L, LDH 403 u/L and haptoglobin <0.1 g/L. A kidney biopsy was taken which showed chronic and active TMA, with 20% glomerulosclerosis and moderate IFTA. After unsuccessful treatment with plasmapheresis, eculizumab was started. This resulted in recovery of TMA parameters but no improvement of kidney function was seen and dialysis was started. Genetic screening revealed a C3 variant. Two years later the patient received a (LUR donor) kidney transplantation. The blood sample for evaluation of C5b-9 deposition was taken 3.8 years after the transplantation.

Patient 17 presented from the age of 22 years with several episodes of severe hypertension and acute kidney injury. TMA parameters were not always evaluated, however elevated LDH (e.g. 476 u/L) and lower thrombocytes (e.g. 178 x 10^9/L) were seen. The episodes were treated with intensive blood

pressure treatment. At the age of 35 years ESRD occurred and hemodialysis was started. Genetic analysis showed a CFH variant, and in retrospect the episodes of hypertensive kidney injury were diagnosed as aHUS. Shortly after start of RRT a (LUR donor) kidney transplantation was performed. The blood sample for evaluation of C5b-9 deposition was taken 3.9 years after the transplantation.

Patient 18 presented at the age of 38 years with AKI and TMA. Relevant laboratory parameters at presentation were: serum creatinine 1730 µmol/L, Hb 5.1 mmol/L, thrombocytes 224 x 10^9/L, LDH 2420 u/L, schistocytes positive. Genetic analysis showed a C3 variant. Hemodialysis was started. The patient received two kidney transplantations which were both lost due to aHUS recurrence. Ten years after initial presentation a third (deceased donor) kidney transplantation was performed. The blood sample for evaluation of C5b-9 deposition was taken 5.1 years after this transplantation.

Patient 19 presented at the age of 24 years with AKI in another country. A kidney biopsy was taken, but the biopsy report was not diagnostic (and the biopsy material could not be revised). There are no further details available. Dialysis was started and two years later a first (LRD donor) kidney transplantation was performed. Three years after transplantation the patient presented with TMA and the graft was lost. Genetic analysis showed no variant. Eight years later a second (deceased donor) kidney transplantation was performed. The blood sample for evaluation of C5b-9 deposition was taken 5.3 years after this transplantation.

Patient 20, with a positive family history of aHUS, presented at the age of 18 years with aHUS after pregnancy. Relevant laboratory values at presentation were: serum creatinine 184 µmol/L, Hb 5.9 mmol/L, thrombocytes 51 x 10^9/L, LDH 2550 u/L, schistocytes positive, ALAT 24 u/L. Genetic analysis showed a C3 variant. Because of ESRD hemodialysis was started. One year later a first (deceased donor) kidney transplantation was performed, which was lost to rejection. Two years later a second (deceased donor) kidney transplantation was performed, which functioned for 38 years, however complicated by multiple rejection episodes and a renal artery stenosis. Four years after graft loss a third (LUR donor) kidney transplantation was performed. The blood sample for evaluation of C5b-9 deposition was taken 5.9 years after this transplantation.

Patient 21 presented at the age of 52 years with ESRD and TMA, and started with hemodialysis. Genetic analysis showed a C3 variant. Two years later the patient received a (LR donor) kidney transplantation. The graft was lost after two years due to aHUS recurrence. Three years later a second (LUR donor) kidney transplantation was performed. The blood sample for evaluation of C5b-9 deposition was taken 6.9 years after this transplantation.

Patient 22 presented at the age of 38 years with AKI and TMA. Relevant laboratory values were: serum creatinine 192 µmol/L, Hb 5.2 mmol/L, thrombocytes 82 x 10^9/L, LDH 2075 u/L. Genetic analysis revealed a C3 variant. Despite blood pressure treatment kidney function deteriorated and hemodialysis was started. Two years later a first (LR donor) kidney transplantation was performed, however after 5 months the graft was lost due to aHUS recurrence. Six years later a second (LUR donor) kidney transplantation was performed. The blood sample for evaluation of C5b-9 deposition was taken 8.5 years after this transplantation.

Patient 23 presented at the age of 28 years with ESRD due to aHUS. Genetic analysis showed a CFH variant. A first pre-emptive (LR donor) kidney transplantation was lost, within 1 year, due to aHUS recurrence. Two years later a second (LR donor) kidney transplantation was performed. The blood sample for evaluation of C5b-9 deposition was taken 8.6 years after this transplantation.

Patient 24 presented at the age of 50 years with ESRD due to aHUS. Genetic analysis showed a C3 variant. Hemodialysis was started and four years later a (LUR donor) kidney transplantation was performed. The blood sample for evaluation of C5b-9 deposition was taken 9.3 years after this transplantation.

Patient 25 presented at the age of 37 years with AKI and TMA. Relevant laboratory values were: serum creatinine 479 µmol/L, Hb 4.7 mmol/L, thrombocytes 144 x 10^9/L. Genetic analysis showed a MCP variant. The patient was treated with plasmapheresis and hemodialysis for several months. Kidney function partially improved but severe CKD remained. Thirteen years later a pre-emptive (LUR donor) kidney transplantation was performed. The blood sample for evaluation of C5b-9 deposition was taken 11.2 years after this transplantation.

Patient 26, with a positive family history of aHUS, presented at the age of 18 years with ESRD and aHUS. Genetic analysis showed a C3 variant. Dialysis was started until (LR donor) kidney transplantation 3 years later. The transplantation was complicated by TMCR which was treated with MPS and ATG. During

this period there was laboratory evidence of TMA and tacrolimus was switched to sirolimus. No additional therapy was given and recovery of TMA parameters and kidney function was seen. The blood sample for evaluation of C5b-9 deposition was taken 11.2 years after this transplantation.

Supplemental Text S2: Review of the literature on the C5b-9 endothelial cell assay

Noris et al introduced the ex vivo assay with measurement of complement deposition on microvascular endothelial cells (of dermal origin) as a test to be used in the clinical arena in 2014.[1] Since then, the test has been further evaluated by the Bergamo group [2, 3], and by investigators from The Netherlands [4-6].

We have analyzed the studies. It is important to know that the investigators evaluated complement deposition on ADP-activated and/or resting endothelial cells; samples were taken from patients in various stages of aHUS (i.e. active disease at presentation or relapse; in remission without therapy or > 6 months after eculizumab withdrawal; in remission while treated with eculizumab), and the majority of the patients had disease in the native kidney. Few patients were studied prospectively, with samples collected regularly during follow-up. Some studies evaluated healthy family members, either known carriers or non-carriers of the variant. Inclusion of patients with "secondary" thrombotic microangiopathy (TMA), and chronic kidney disease (CKD) controls has been variable.

An overview is given in the Tables. Supplementary Table S6 provides clinical characteristics of patients with "active" aHUS and the percentage of positive test results on resting and activated endothelial cells (when available). These patients were diagnosed clinically and mostly characterized by acute kidney injury (AKI) and laboratory features of microangiopathic hemolytic anemia (MAHA). In addition, results of samples tested in (often other) patients with known aHUS in remission without therapy, and during therapy with eculizumab are provided. It is evident that the assay was positive in all evaluable aHUS patients with active disease, whether using resting (70/70; 100%) or activated endothelial cells (74/74; 100%). Thus, sensitivity of the test for aHUS might be 100%. As expected the test was negative in samples that contained eculizumab. More interesting are the measurements using samples of patients in remission without therapy: C5b-9 deposition on resting endothelial cells was increased in only 3/38 cases; in contrast, when using activated endothelial cells the test remained positive in all but one patient. This finding of increased C5b-9 deposition on activated endothelial cells in aHUS patients in remission stimulated further research trying to assess the potential role of the assay to identify the presence of a genetic cause of complement dysregulation.[3]

Although sensitivity is important, tests used in the setting of very rare diseases (e.g. low population prevalence), which must be differentiated from more prevalent disease mimickers, must have high specificity. Therefore, studies should evaluate the performance of the assay not only in patients with CKD or dialysis, but especially in patients with TMA due to other causes. Unfortunately, the number of patients with secondary TMA included in the studies is limited, and clinical details are often lacking. Supplementary Table S7 summarizes the test results in patients with systemic secondary TMA, and patients with may have clinical features that may partly overlap with aHUS (e.g. pregnancy associated TMA or malignant hypertension).[2, 6] The data provided by Timmermans et al. are discussed separately (see Supplementary Table S8 and text below), since these investigators used the positive test to define complement mediated TMA. Bias was introduced (a form of circular reasoning) since specificity of a test to diagnose aHUS cannot be evaluated if the test is used a diagnostic criterium.[6] From Supplementary Table S7 it is evident that numbers of patients per condition is small. Still, the data indicate that, while patients with CKD, or dialysis disclose negative tests, many patients with secondary TMA demonstrate increased C5b-9 deposition. The study of Galbusera et al. indicates that this conclusion holds for tests using either resting or activated endothelial cells. Of note, Galbusera et al showed that the test remained positive in a substantial number of patients with secondary TMA who are in remission, most notable when using activated endothelial cells (test positive on resting endothelial cells in 2/9 cases and when using active endothelial cells in 7/10 cases respectively). In theory, some of these patients might have an underlying complement abnormality explaining positivity on activated endothelium, however most patients with secondary TMA were genetically evaluated and a variant of unknown significance was found in only 2 of the 28 patients.[2] Importantly, the study does not provide information regarding the duration of remission and the interval between active disease and subsequent sampling. Spanish researchers also found elevated C5b-9 deposits in patients with secondary forms of TMA (e.g. drug induced TMA, HELLP, pre-eclampsia) and in patients with systemic inflammation (e.g. sepsis, SIRS, COVID). Admittedly, their assay was performed with modifications: activated endothelial cells, and activated plasma (patient plasma was added to control sera) was used, possibly affecting comparability with the C5b-9 assay, as developed by Noris et al..[7-9]

We separately discuss the studies of Timmermans et al.[4-6] The latest manuscript published in 2021 [6] provides an overview of new and previously published patients.[4, 5] The investigators included mostly patients with histologically defined TMA, in the absence of systemic MAHA. Thus, only 41% of patients had systemic hemolysis, clearly different from the patient cohorts included in the Italian and Spanish studies (see Supplementary Table S7). More importantly, the authors used the endothelial assay to diagnose complement mediated aHUS, thus using the test as diagnostic tool. A detailed analysis of

the study questions the validity. Patients were also classified as primary and secondary HUS using the clinical criteria as proposed by KDIGO. Thus, three subgroups could be defined: 1. Patients with clinical aHUS and a positive endothelial test, 2. Patients with a secondary cause and a positive test (so called "complement mediated TMA"), and 3. Patients with a secondary cause and a negative test (non-complement mediated TMA). Supplementary Table S8 illustrates the differences between the three groups. It is evident that patients with clinical aHUS closely resemble the "typical" aHUS patients as reported in literature i.e. the majority having systemic hemolysis, low thrombocytes, and a known complement defect in 70% of patients. In contrast, only 38% of patients with non-complement TMA showed systemic hemolysis, and a genetic variant was found in none. Interestingly, the patients in the second group, with clinical secondary HUS but defined as C-TMA, clearly differ from the patients with clinical aHUS: systemic hemolysis was observed in only 29% of patients, thrombocytes were often normal, the majority needed dialysis at presentation, and only 35% of patients had a genetic variant. In our opinion, this data suggest that the patients with C-TMA are a heterogeneous group of patients, all with a defined trigger, some with complement dysregulation (validating a diagnosis of aHUS) and others without (pure secondary TMA). In the absence of a validated diagnostic biomarker, it is impossible to draw conclusions. The authors suggest that there was an association between eculizumab use and outcome in the second group. If true and confirmed, the test could be valuable to allow cost-effective use of eculizumab. However, other authors showed that a positive test does not always equal need for eculizumab therapy. Patients with STEC-HUS are the best example, in these patients the test is positive, however the efficacy of eculizumab is not demonstrated (likely because most patients with STEC-HUS recover due to the presence of an intact complement regulatory system).[10-13] In the study of Timmermans, there were 31 patients (diagnosed by the authors) with C-TMA, and of these 14 patients, not responding to standard of care, received eculizumab. Response was evaluated in 30 patients. End-stage renal disease (ESRD) was observed in 2/14 patients treated with eculizumab and in 14/16 patients treated without eculizumab. Although this suggests benefit, the data do not allow conclusions: the study was retrospective, and treatment not randomized. Clearly, there are major differences between patients treated with eculizumab vs non-treated: patients who received eculizumab had lower thrombocytes (median 90 vs 138 x 10^9/L), more preserved kidney function (serum creatinine 492 vs 854 µmol/l; a lower percentage of patients on dialysis 57% vs 88%), and unexpectedly a lower prevalence of pathogenic variants (21% vs 50%). Thus, the better outcome in patients with eculizumab likely reflects selection bias. It should also be noted that in the patients with non-complement mediated TMA (of which 5 received eculizumab!) ESRD developed in 5/16 patients treated with conservative therapy only, thus not significantly different from the Eculizumab treated C-TMA patients (2/14). The authors mention that non-responding

patients more often presented with anuria/oliguria, serum creatinine > 500 µmol/l, and > 50% IFTA on kidney biopsy, indicating that outcome (or non-response) were mainly dictated by the chronicity of the disease at presentation, which suggests a longstanding history of kidney disease prior to presentation.

Obviously, it would be of great importance if the test would allow to predict a relapse. Galbusera et al. reported 22 aHUS patients, in whom C5b-9 deposition on resting cells was monitored after discontinuation or tapering of eculizumab. A relapse was observed in 5/6 patients who showed increased activity on resting cells. In contrast, C5b-9 deposition remained normal in 16 patients; all stayed in remission. However, it not evident that the test can be used to *predict* relapse. In fact, in all 5 patients diagnosed with relapse abnormalities in thrombocytes, LDH, haptoglobin and/or serum creatinine were already notable when elevated C5b-9 deposition was found (Supplementary Table S8).

Supplementary Table S6: Clinical characteristics of patients with active aHUS at disease onset and results of the C5b-9 assay

Author	Noris 2014[1]	Galbusera 2019[2]	Gastoldi 2023[3]	Timmermans 2021[6]
**Patients with active aHUS**	14	52	16	13
Age (years)	Unknown	Mean 28 (range 0.5-90)	Mean 16 (range 0.6-48)	Mean 30 ± 25
Gender (M/F)	Unknown	21M/31F	8M/8F	5M/8F
Systemic hemolysis	Inclusion criteria AKI + MAHA	Inclusion criteria AKI + MAHA	Inclusion criteria AKI + MAHA	9/13 (69.2%)
Thrombocytes	Unknown	Mean 83 x 10^9/L ± 50	Unknown	Median 36 x 10^9/L (IQR 12-200)
LDH	Unknown	Mean 2203 U/L ± 1886	Unknown	Median 1251 U/L (IQR 711-2390)
AKI	Unknown	Likely yes	Unknown	Unknown
Creatinine	Unknown	Mean 539 µmol/L ± 486	Unknown	Median 321 µmol/L (IQR 193-407)
Genetic variant (%)	3/14 (21.4%)	19/45 (42.2%)	15/16 (93.8%)	9/13 (70%)
Anti CFH Ab (%)	1/14 (7.1%)	Unknown	0%	Unknown
**Kidney Tx patients included (%)**	1 (2.8%)	Unknown	0%	0%
	**RESTING Endothelium: positive/total N (%)**			
**aHUS active**	13/13 (100%)	46/46 (100%)	n.a.	11/11 (100%)
**aHUS remission without therapy**	1/6 (1.7%)	2/32 (6.3%)	n.a.	n.a
**aHUS remission with ECU**	n.a.	0/26 (0%)	n.a.	n.a.
	**ACTIVATED Endothelium: positive/total N (%)**			
**aHUS active**	9/9 (100%)	49/49 (100%)	16/16 (100%)	n.a.
**aHUS remission without therapy**	29/29 (100%)	59/59 (100%)	49/50 (98%)	n.a.
**aHUS remission with ECU**	0/4 (0%)	0/37 (0%)	0/4 (0%)	n.a.

Remarks: Noris, Gastoldi and Galbusera represent the Italian investigators. These authors defined aHUS as AKI+MAHA. Although the focus of the manuscripts differed, their patients likely overlap. Timmermans included patients described in earlier reports.[5, 14]

Abbreviations: AKI: acute kidney injury, anti CFH Ab: anti factor H auto-antibodies, ECU: eculizumab, kidney Tx: kidney transplantation, n.a.: not applicable

Supplemental Table S7: Results of C5b-9 assay on resting endothelium in patients with secondary TMA

Author	Galbusera 2019[2]	Timmermans 2021[6]
**Sec TMA (active disease)**	15/15 (100%) #	
**Sec TMA (in remission without therapy)**	2/9 (22.2%)	
**STEC-HUS**	5/5 (100%)	
**HELLP**		0/3 (0%)
**Pregnancy associated TMA**		8/8 (100%)
**Hypertensive emergency**		18/30 (60%)
**De novo TMA after Ktx**		2/5 (40%)
**DITMA**		0/2 (0%)
**Streptococcal HUS**		1/1 (100%)
**Postsurgical TMA**		2/3 (67%)
**CKD**	0/5 (0%)	
**Dialysis**	0/7 (0%)	

secondary TMA associated with systemic lupus erythematosus , multiple myeloma, lymphoma – chemotherapy, bone marrow transplantation, graft versus host disease, post kidney transplantation, focal segmental glomerulosclerosis, membranoproliferative glomerulonephritis, malignant nephroangiosclerosis, acute post-infectious glomerulonephritis, IgA nephropathy, malignant hypertension, rheumatoid arthritis, thyroiditis, Wegener granulomatosis, multiple sclerosis.

Abbreviations: CKD: chronic kidney injury, DITMA; drug induced TMA; HELLP: hemolysis, elevated liver enzymes, low platelet count, Ktx: kidney transplantation, sec TMA: secondary TMA, STEC-HUS: Shiga toxin producing Escherichia Coli hemolytic uremic syndrome, TMA: thrombotic microangiopathy.

Supplementary Table S8: Comparison of clinical diagnosis and diagnosis based on C5b-9 assay as reported by Timmermans et al.

**Group**	**1**<br>**n=13**	**2**<br>**n=31**	**3**<br>**n=21**
**Clinical diagnosis**	**aHUS**	**secondary TMA**	**secondary TMA**
**C5b-9 assay**	**+**	**+**	**-**
**Authors' diagnosis**	C-TMA	C-TMA	Non-complement mediated TMA
**Age (years)**	30 ± 25	38 ± 13	42 ± 13
**Gender M/F**	5M / 8F	14M / 17F	12M / 9F
**Systemic hemolysis**	9 (69%)	9 (29%)	8 (38%)
**Thrombocytes (x10^9/L)**	36 (12-200)	133 (75-228)	95 (52-178)
**LDH (U/L)**	1251 (711-2390)	680 (305-1486)	762 (465-1222)
**AKI**	Unknown*	Unknown*	Unknown*
**Screat (µmol/L)**	321 (193-407)	561 (356-1065)	485 (231-778)
**Dialysis**	5 (38%)	22 (71%)	11 (52%)
**Genetic variant (%)**	9 (70%)	11 (35%)	0 (0%)
**Eculizumab therapy**	5	14	5

Adapted from Timmermans et al.[6] In this study patients with TMA were diagnosed as aHUS or secondary TMA using well defined international clinical and laboratory criteria. These patients were also defined by the authors as complement mediated TMA (C-TMA) versus non-complement mediated TMA based on the results of the C5b-9 endothelial cell assay. Finally, three groups were discerned: clinical aHUS, assay positive; clinical secondary TMA, assay positive, clinical secondary TMA, assay negative. Age was expressed as mean ± SD, other results were expressed as median, IQR.

See text for further information.

*It is unlikely that all patients had AKI, since there were many patients with >50% interstitial fibrosis and tubular atrophy on kidney biopsy.

Abbreviations: AKI: acute kidney injury, F: female, IQR: interquartile range, M: male, sec TMA: secondary thrombotic microangiopathy

Supplementary Table S9: Clinical parameters of the 5 aHUS patients reported by Galbusera et al. at the time of elevated C5b-9 deposition on resting HMECs

Case	T (< 150x10^9/L)	LDH (> 500 IU/L)	Hapto (< 49 mg/dL)	AKI *	C5b-9 resting HMECs (>150%)
**1**	416 x10^9/L	**1027**	ND	N	**233**
**2**	348 x10^9/L	**539**	**<1**	N	**209**
**3**	210 x10^9/L	**513**	**9**	N	**188**
**4**	203 x10^9/L	220	**33**	**Y**	**172**
**5**	**141 x10^9/L**	**525**	57	N	**442**

* AKI is defined as increase in serum creatinine > 50%, in 7 days.

Abnormal values are in bold. Abbreviations: AKI: acute kidney injury, hapto: haptoglobin, N: No, ND: not done, T: thrombocytes, Y: yes.

References:

1. Noris, M., et al., *Dynamics of complement activation in aHUS and how to monitor eculizumab therapy.* Blood, 2014. **124**(11): p. 1715-26.
2. Galbusera, M., et al., *An Ex Vivo Test of Complement Activation on Endothelium for Individualized Eculizumab Therapy in Hemolytic Uremic Syndrome.* Am J Kidney Dis, 2019. **74**(1): p. 56-72.
3. Gastoldi, S., et al., *An ex vivo test to investigate genetic factors conferring susceptibility to atypical haemolytic uremic syndrome.* Front Immunol, 2023. **14**: p. 1112257.
4. Timmermans, S., et al., *C5b9 Formation on Endothelial Cells Reflects Complement Defects among Patients with Renal Thrombotic Microangiopathy and Severe Hypertension.* J Am Soc Nephrol, 2018.
5. Timmermans, S., et al., *Diagnostic and Risk Factors for Complement Defects in Hypertensive Emergency and Thrombotic Microangiopathy.* Hypertension, 2020. **75**(2): p. 422-430.
6. Timmermans, S., et al., *Functional and Genetic Landscape of Complement Dysregulation Along the Spectrum of Thrombotic Microangiopathy and its Potential Implications on Clinical Outcomes.* Kidney Int Rep, 2021. **6**(4): p. 1099-1109.
7. Blasco, M., et al., *Complement as the enabler of carfilzomib-induced thrombotic microangiopathy.* Br J Haematol, 2021. **193**(1): p. 181-187.
8. Palomo, M., et al., *Complement Activation and Thrombotic Microangiopathies.* Clin J Am Soc Nephrol, 2019. **14**(12): p. 1719-1732.
9. Fernández, S., et al., *Distinctive Biomarker Features in the Endotheliopathy of COVID-19 and Septic Syndromes.* Shock, 2022. **57**(1): p. 95-105.
10. Percheron, L., et al., *Eculizumab treatment in severe pediatric STEC-HUS: a multicenter retrospective study.* Pediatr Nephrol, 2018. **33**(8): p. 1385-1394.
11. Menne, J., et al., *Validation of treatment strategies for enterohaemorrhagic Escherichia coli O104:H4 induced haemolytic uraemic syndrome: case-control study.* Bmj, 2012. **345**: p. e4565.
12. Kielstein, J.T., et al., *Best supportive care and therapeutic plasma exchange with or without eculizumab in Shiga-toxin-producing E. coli O104:H4 induced haemolytic-uraemic syndrome: an analysis of the German STEC-HUS registry.* Nephrol Dial Transplant, 2012. **27**(10): p. 3807-15.
13. Garnier, A., et al., *Efficacy and Safety of Eculizumab in Pediatric Patients Affected by Shiga Toxin-Related Hemolytic and Uremic Syndrome: A Randomized, Placebo-Controlled Trial.* J Am Soc Nephrol, 2023.
14. Timmermans, S., et al., *C5b9 Formation on Endothelial Cells Reflects Complement Defects among Patients with Renal Thrombotic Microangiopathy and Severe Hypertension.* J Am Soc Nephrol, 2018. **29**(8): p. 2234-2243.
